# Low Proportion of Linezolid and Daptomycin Resistance Among Bloodborne Vancomycin-Resistant *Enterococcus faecium* and Methicillin-Resistant *Staphylococcus aureus* Infections in Europe

**DOI:** 10.3389/fmicb.2021.664199

**Published:** 2021-05-31

**Authors:** Robby Markwart, Niklas Willrich, Tim Eckmanns, Guido Werner, Olaniyi Ayobami

**Affiliations:** ^1^Jena University Hospital, Institute of General Practice and Family Medicine, Jena, Germany; ^2^Unit 37: Nosocomial Infections, Surveillance of Antimicrobial Resistance and Consumption, Robert Koch Institute, Berlin, Germany; ^3^Division Nosocomial Pathogens and Antibiotic Resistances, Department of Infectious Diseases, National Reference Centre for Staphylococci and Enterococci, Robert Koch Institute, Wernigerode, Germany

**Keywords:** vancomycin-resistant *Enterococcus faecium*, methicillin-resistant *Staphylococcus aureus*, daptomycin, linezolid, last-resort antibiotics, antimicrobial resistance

## Abstract

Vancomycin-resistant *Enterococcus faecium* (VREF) and methicillin-resistant *Staphylococcus aureus* (MRSA) are associated with significant health burden. We investigated linezolid and daptomycin resistance among VREF and MRSA in the EU/EEA between 2014 and 2018. Descriptive statistics and multivariable logistic regression were used to analyze 6,949 VREF and 35,131 MRSA blood isolates from patients with bloodstream infection. The population-weighted mean proportion of linezolid resistance in VREF and MRSA between 2014 and 2018 was 1.6% (95% CI 1.33–2.03%) and 0.28% (95% CI 0.32–0.38%), respectively. Daptomycin resistance in MRSA isolates was similarly low [1.1% (95% CI 0.75–1.6%)]. On the European level, there was no temporal change of daptomycin and linezolid resistance in MRSA and VREF. Multivariable regression analyses showed that there was a higher likelihood of linezolid and daptomycin resistance in MRSA (aOR: 2.74, *p* < 0.001; aOR: 2.25, *p* < 0.001) and linezolid in VREF (aOR: 1.99, *p* < 0.001) compared to their sensitive isolates. The low proportion of linezolid and daptomycin resistance in VREF and MRSA suggests that these last-resort antibiotics remain effective and will continue to play an important role in the clinical management of these infections in Europe. However, regional and national efforts to contain antimicrobial resistance should continue to monitor the trend through strengthened surveillance that includes genomic surveillance for early warning and action.

## Introduction

*Staphylococcus aureus* and *Enterococcus faecium* are Gram-positive pathogens that frequently colonize the skin, nostrils, and gut of humans with potential for invasive infections in humans (Fisher and Phillips, 2009). They are the most important Gram-positive organisms causing nosocomial infections especially bloodstream and other invasive infections (Suetens et al., 2018). These pathogens have shown combined resistance to multiple antimicrobial classes including beta-lactams, cephalosporins, fluoroquinolones etc. making their treatment increasingly difficult (Hollenbeck and Rice, 2012; O’Driscoll and Crank, 2015; Lakhundi and Zhang, 2018). Consequently, they are responsible for significant health and economic burden on hospitalized patients, families and society (Zhen et al., 2019).

In Europe and many other countries across the world, *Enterococcus spp.* and *S. aureus* infections are commonly observed among hospital patients with high vancomycin and methicillin resistance proportions, respectively (Lakhundi and Zhang, 2018; García-Solache and Rice, 2019). Moreover, these multidrug-resistant pathogens have been associated with significant mortality and morbidity in several healthcare settings (Caballero-Granado et al., 2001; Pinholt et al., 2014; Boncagni et al., 2015; Brady et al., 2017; Nelson et al., 2017; Kramer et al., 2018; Cassini et al., 2019). Across Europe, the population-weighted proportion of methicillin resistance among *S. aureus* is 16.4%, while vancomycin resistance among *E. faecium* isolates was estimated to be 18.3% in 2019 (Ayobami et al., 2020a; European Centre for Disease Prevention Control, 2020). Recent studies have demonstrated the rising trend of vancomycin resistance in *Enterococcus spp*., especially in *E. faecium*, and its pervasiveness across Europe (Markwart et al., 2019; Ayobami et al., 2020a).

While a declining trend of MRSA is reported in Europe, the incidence is increasing among the extremes of age groups, and its resistance proportion is still high in many countries of the European Union (Cassini et al., 2019; European Centre for Disease Prevention Control, 2020). The diminishing antibiotic arsenal, widespread resistance to several antibiotics, and other pharmacological concerns, renders antimicrobials such as linezolid and daptomycin, the antibiotics of last-resort to manage VREF and MRSA infection especially among critically ill hospitalized patients who are at the greatest risk of mortality (Rodvold and McConeghy, 2014; Hashemian et al., 2018; Heidary et al., 2018). Despite the therapeutic utility of these drugs, there is an increasing report of linezolid, and daptomycin resistance among patients infected with VREF and MRSA worldwide (Miller et al., 2016; Bender et al., 2018a; Heidary et al., 2018).

Ensuring the effectiveness of these last resort antibiotics is a priority globally, considering the severity of multi-drug resistant Gram-positive bacterial infections, broken antibiotic market and the prevailing insufficient public health actions to address antimicrobial resistance. It is on this premise that this study sought to characterize the magnitude and trend of resistance of VREF and MRSA to linezolid and daptomycin using European-wide surveillance data (2014-2018) from patients with bloodstream infections.

## Materials and Methods

### Outcomes, Study Design, and the European Antimicrobial Resistance Surveillance Database

The primary outcome was the population-weighted proportion of linezolid resistance among vancomycin-resistant *E. faecium*, and resistance to linezolid and daptomycin among methicillin-resistant *S. aureus* isolates. We conducted a retrospective observational study on *E. faecium* and *S. aureus* (2014–2018) using data retrieved from the European Antimicrobial Resistance Surveillance Network (EARS-Net) TESSy database with the approval of the European Centre for Disease Prevention and Control. EARS-Net is a network of European surveillance systems that collects routine clinical antimicrobial susceptibility (AST) data on invasive isolates [blood and cerebrospinal fluid (CSF)] from the 27 countries in the European Union as well as Norway, Iceland, and the United Kingdom (European Centre for Disease Prevention Control, 2018a).

### Selection of Isolates

The TESSy database of EARS-Net only includes the first isolate from a given patient in the respective year. To identify unique isolates, we created a composite identifier for each isolate using “R” (v. 3.6.1). The composite identifier is composed of the (i) reporting country, (ii) unique laboratory identifier, (iii) hospital identifier, (iv) patient identifier, (v) date of sample collection, and (vi) the identified pathogen. These variables are part of the original dataset from EARS-Net and are provided by the participating countries. Isolates with duplicate composite identifiers, more than one AST against the same antibiotic, and those not assigned a hospital ID were excluded. Only *E. faecium* isolates that were tested for vancomycin susceptibility were included. For *S. aureus*, only isolates that were tested for “methicillin resistance” (see the section on *Variables and Definitions* below) were included for analysis.

### Variables and Definitions

Patient age was categorized into four age categories (<1, 1–19, 20–64, ≥65 years). Patient gender was classified into a female, male or unknown. The country of origin of the isolate was grouped into four major regions of Europe (**North:** Denmark, Finland, Iceland, Ireland, Norway, Sweden, United Kingdom; **West:** Austria, Belgium, France, Germany, Luxembourg, Netherlands; **South:** Croatia, Cyprus, Greece, Hungary, Italy, Malta, Portugal, Slovenia, Spain; **East:** Bulgaria, Czech Republic, Estonia, Latvia, Lithuania, Poland, Romania, Slovakia). Hospital unit types were categorized into intensive care units (ICU) (including pediatric ICUs) and non-intensive care units, such as internal medicine, surgery, oncology, etc.

In the TESSy database, results on antimicrobial susceptibility testing are classified as sensitive (S), intermediate (I), or resistant (R) based on the standards used in the participating laboratories, e.g., guidelines and annual tables of clinical breakpoints of the European Committee on Antimicrobial Susceptibility Testing [EUCAST vs. 4.0 (2014) to 8.1 (2018)], Clinical and Laboratory Standards Institute (CLSI) or other national guidelines. An *Enterococcus faecium* isolate was defined as vancomycin-resistant if it was tested resistant or intermediate against vancomycin based on the MIC cut-offs in the respective AST guideline used by the laboratories and validated by EARS-net. Even though EARS-Net prioritized detection of the *mecA* gene by PCR or positive PBP2A-agglutination test over phenotypic susceptibility results, *S. aureus* isolates were defined as methicillin-resistant if it was tested resistant or intermediate against oxacillin or cefoxitin. An isolate was defined as resistant to linezolid and daptomycin if it was tested as resistant or intermediate against those antibiotics, respectively.

### Outcomes and Statistical Analyses

The population-weighted proportion of antibiotic-resistant *E. faecium* and *S. aureus* among all tested isolates was expressed as a percentage with its 95% confidence intervals (95% CI). To assess the potential association of last-resort antibiotic resistance with ward type (i.e., ICU vs. non-ICU) and year of sampling (temporal trend), respectively, multivariable logistic regression analyses were performed including the following independent variables: Year of sampling, gender, age group, European region, and ward type. These variables were selected before the analysis based on the availability of data and our prior hypotheses about variables that may be associated with linezolid and daptomycin resistance in VREF and MRSA. All variables were treated as categorical variables, except year of sampling which was treated as a continuous variable. All statistical analyses were performed using R version 3.6.1 (R Core Team, 2019) and the “survey” package (version 3.37) (Lumley, 2020). For all analyses in all strata (i.e., descriptive analyses of resistance proportions, logistic regression analyses, and Chi^2^-tests) we accounted for clustering at hospital level and applied European region population-based weighting (Ayobami et al., 2020b). The population of the four European regions was calculated from the individual populations of the region’s countries. Population data of individual countries were obtained from the Eurostat database (Eurostat, 2020). European regions population weighting was used to ensure that the data from each major European region contributed proportionally (in relation to their respective population size) to the calculation of resistance proportions. This was done to minimize bias from significant differences in isolate numbers from various European regions.

## Results

### Baseline Characteristics

In total, 55,074 *E. faecium* and 211,379 *S. aureus* isolates from patients with bloodstream infections were included in the analysis. The characteristics of the included isolates are outlined in Table 1. The population-weighted mean proportion (2014–2018) of vancomycin resistance among *E. faecium* isolates was 13.1% (95% CI 11.5–14.7%) and 17.3% (95% CI 16.1–18.5%) of the *S. aureus* isolates were methicillin-resistant. The proportional distribution of the isolates from the regions roughly corresponded with the population size of the four European regions, although isolates from Eastern Europe were somewhat underrepresented in the dataset. For both pathogens, isolates were mainly derived from elderly patients [*E. faecium*: median = 70 years, interquartile range (IQR) = 60–79 years; *S. aureus*: median = 70 years, IQR = 57–80 years] and from male patients (*E. faecium*: female/male ratio: 0.64; S. aureus: female/male ratio: 0.61). Among the *E. faecium* and *S. aureus* isolates, 24.7% and 12.8% were collected in ICUs, respectively.

**TABLE 1:** Baseline characteristics of analyzed isolates of *Enterococcus faecium* and *Staphylococcus aureus* from patients with bloodstream infections.

	*E. faecium*		*S. aureus*	
*Number of isolates (%)*	55,074	(100)	211,379	(100)
*Number of isolates tested against linezolid (% tested)*	43,406	(78.8)	165,461	(78.3)
*Number of isolates tested against daptomycin (% tested)*	–		44,197	(20.9)
Number of VREF/MRSA isolates (%*)	6949	(13.1*)	35,131	(17.3*)
***Year of sampling***				
2014 (n, %)	8319	(15.1)	32,741	(15.5)
2015 (n, %)	9155	(16.6)	36,846	(17.4)
2016 (n, %)	12,069	(21.9)	45,312	(21,4)
2017 (n, %)	12,089	(22.0)	46,759	(22.1)
2018 (n, %)	13,442	(24.4)	49,721	(23.5)
***European regions***				
North (n, %)	14,237	(25.9)	35,644	(16.9)
West (n, %)	20,593	(37.4)	101,500	(48.0)
South (n, %)	15,278	(27.7)	61,976	(29.3)
East (n, %)	4966	(9.0)	12,259	(5.8)
***Gender of patients***				
Female (n, %)	19,785	(32.8)	74,006	(35.0)
Male (n, %)	30,695	(58.8)	121,780	(57.6)
NA (n, %)	4594	(8.5)	15,593	(7.4)
Sex ratio (f/m)	0.64		0.61	
***Age of patients***				
<1 year (n, %)	562	(1.0)	4389	(2.1)
1–19 years (n, %)	721	(1.3)	6005	(2.8)
20–59 years (n, %)	16,850	(30.6)	65,176	(30.8)
≥65 years (n, %)	33,502	(60.8)	125,486	(59.4)
NA (n, %)	3439	(6.2)	10,323	(4.9)
Age (median, IQR)	70yrs	60–79 years	70yrs	57–80 years
***Hospital unit type***				
Intensive care unit	13,606	(24.7)	26,965	(12.8)
Non-intensive care unit	30,802	(55.9)	153,855	(72.8)
NA (n, %)	10,666	(19.4)	30,559	(14.5)
***Number of hospitals***	1873		1955	

***E. faecium,** Enterococcus faecium; **IQR,** Interquartile range; **MRSA,**: Methicillin-resistant Staphylococcus aureus, **S. aureus,** Staphylococcus aureus; **VREF,** vancomycin-resistant Enterococcus faecium proportion.*

### Linezolid Resistance in Vancomycin-Resistant *Enterococcus faecium*

**FIGURE 1:**
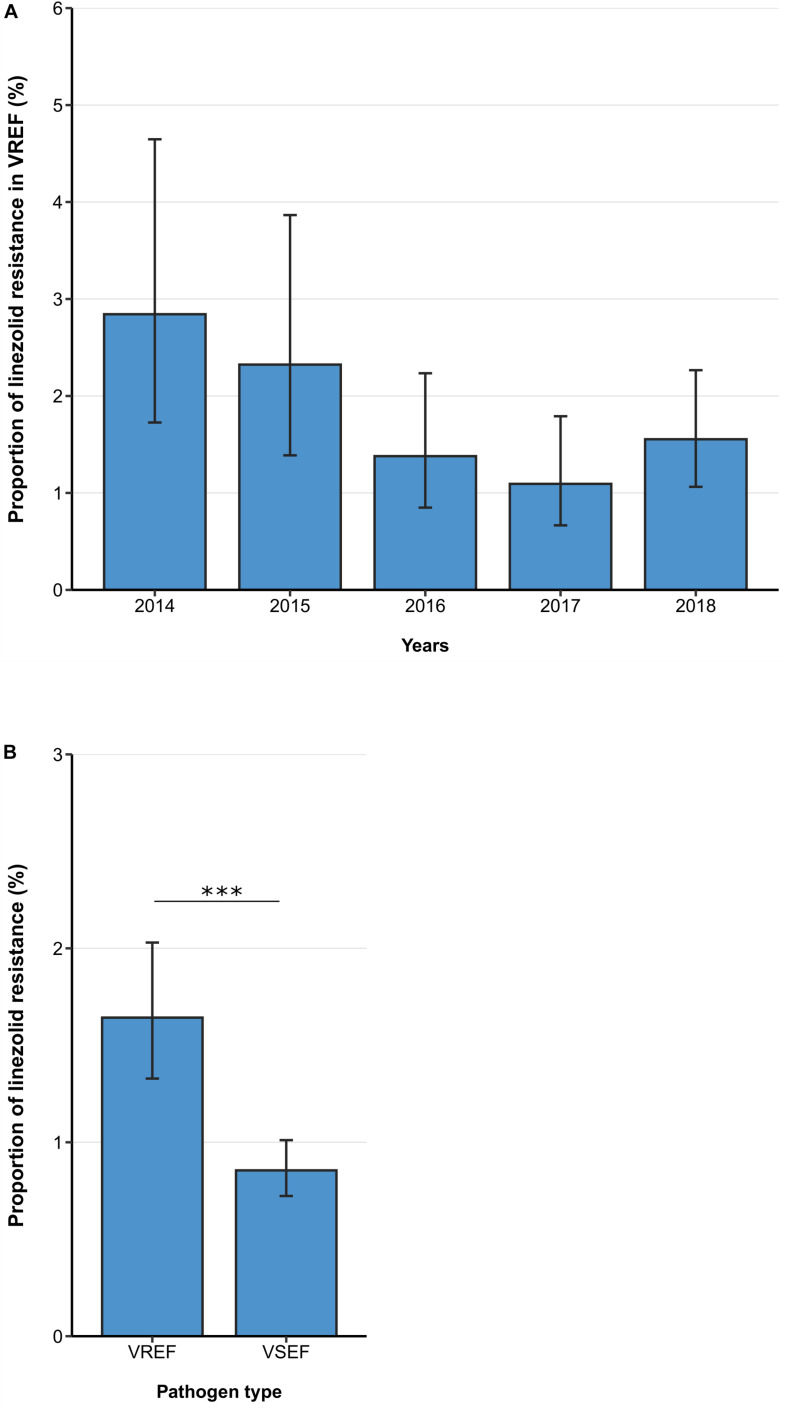
Linezolid resistance in vancomycin-resistant and -sensitive *E. faecium* isolates from patients with bloodstream infections. **(A)** Linezolid resistance proportions in vancomycin-resistant *E. faecium* (VREF) between 2014 and 2018 and **(B)** linezolid resistance in VREF and vancomycin-sensitive *E. faecium* (VSEF) Linezolid resistance proportions are expressed as population-weighted mean proportions of resistant isolates among all tested isolates with corresponding 95% confidence intervals. The difference in linezolid resistance proportions between VREF and VSEF isolates **(B)** was analyzed using the Chi square test. *** *p* < 0.001.

Between 2014 and 2018, the linezolid resistance proportion among vancomycin-resistant *E. faecium* isolates from patients with bloodstream infections was 1.64% (95% CI 1.33–2.03%). Although descriptive analyses of linezolid resistance proportions in VREF isolates suggested a decreasing trend between 2014 and 2018, a multivariable analysis adjusting for factors that might be associated with linezolid resistance did not confirm this declining trend [Odds Ratio (OR) = 0.86 (95%CI 0.73–1.02), *p* = 0.088] (Figure 1A, Table 2). Across all hospital wards, linezolid resistance was lower in vancomycin-sensitive *E. faecium* isolates compared to VREF isolates [0.86% (95% CI 0.72–1.01%) vs. 1.64% (95% CI 1.34–2.03%), *p* < 0.001] (Figure 1B). This finding is supported by the multivariable regression analysis that showed that linezolid resistance more likely occurred in VREF isolates compared to vancomycin-sensitive *E. faecium* isolates [adjusted OR: 1.99 (95% CI 1.56–2.54), *p* < 0.001] (Supplementary Table 1). We found that there was a trend of higher linezolid resistance proportions among VREF isolates in ICUs compared to non-ICUs, although this difference was not statistically significant [1.94% (95% CI 1.31–2.86%) vs. 1.36%, (95% CI 1.03–1.80%), *p* = 0.107; adjusted OR: 1.36 (95% CI 0.87–2.12), *p* = 0.174] (Table 2).

**TABLE 2:** Multivariable logistic regression analysis of factors associated with linezolid resistance in vancomycin-resistant *E. faecium* blood isolates.

		Multivariable analysis		
		*OR*	*(95% CI)*	*p-value*
**Year of sampling (per 1 year increase)**				
	2014–2018	0.86	(0.73–1.02)	0.088
**Unit type**				
	Non-ICU	1	–	–
	ICU	1.36	(0.87–2.12)	0.174
	Unknown	2.12	(0.93–4.87)	0.076
**European region**				
	Eastern	1	–	–
	Northern	0.41	(0.18–0.93)	0.033
	Western	0.84	(0.44–1.62)	0.600
	Southern	0.87	(0.51–1.48)	0.613
**Patient age**				
	<1 year	1	–	–
	1–19 years	0.49	(0.09–2.57)	0.402
	20–64 years	0.25	(0.08–0.79)	0.018
	≥65 years	0.16	(0.05–0.50)	0.001
	Unknown	0.63	(0.18–2.18)	0.464
**Patient gender**				
	Female	1	–	–
	Male	1.37	(0.86–2.19)	0.180
	Unknown	1.26	(0.70–2.29)	0.443

***OR:** Odds Ratio; **CI:** Confidence Interval; **ICU:** Intensive Care Unit.*

### Linezolid Resistance in Methicillin-Resistant *Staphylococcus aureus*

**FIGURE 2:**
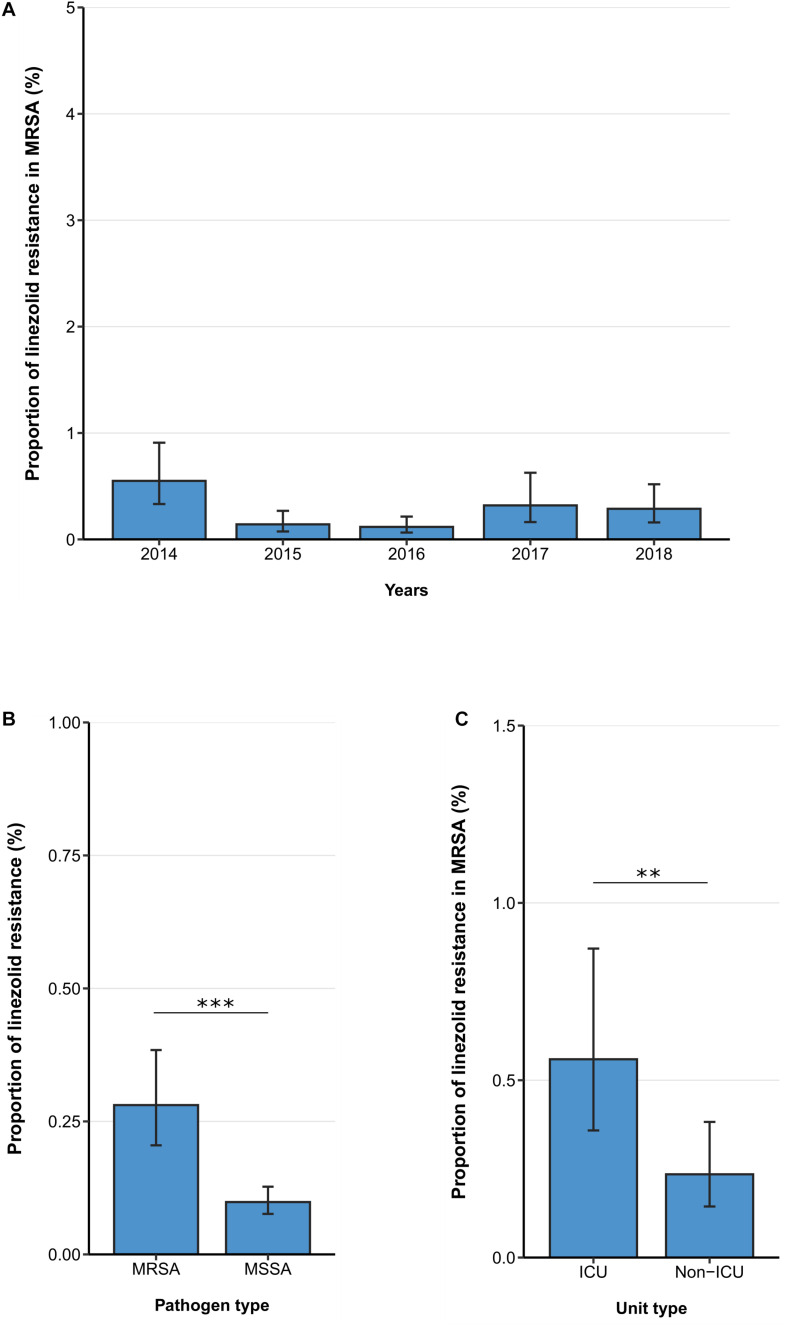
Linezolid resistance in methicillin-resistant and -sensitive *S. aureus* isolates from patients with bloodstream infections. **(A)** Linezolid resistance proportions in methicillin-resistant *S. aureus* (MRSA) between 2014 and 2018, **(B)** linezolid resistance in MRSA and methicillin-sensitive *S. aureus* (MSSA) and **(C)** linezolid resistance in MRSA stratified by unit type (ICU vs. non-ICU). Linezolid resistance proportions are expressed as population-weighted mean proportions of resistant isolates among all tested isolates with corresponding 95% confidence intervals. **(B)** The difference in linezolid resistance proportions between MRSA and MSSA isolates and **(C)** between ICU vs. non-ICU MRSA isolates was analyzed using the Chi square test. ** *p* < 0.01; *** *p* < 0.001.

The linezolid resistance proportion among MRSA isolates from patients with bloodstream infections was 1ow [0.28% (95% CI 0.21–0.38%)] and no temporal change was observed between 2014 and 2018 [adjusted OR: 0.90 (95% CI 0.70–1.16), *p* = 0.410] (Figure 2A). Similar to findings in VREF isolates, hospital-wide linezolid resistance proportion is higher in MRSA isolates compared to methicillin-sensitive *S. aureus* isolates [0.28% (95% CI 0.21–0.38%) vs. 0.10 (95%CI 0.08–0.13%), *p* < 0.001] (Figure 2B). In line with this, the multivariable analysis also showed that the likelihood of linezolid resistance was higher in MRSA isolates than in methicillin-sensitive *S. aureus* isolates [adjusted OR: 2.74 (95% CI 1.98–3.78), *p* < 0.001] (Supplementary Table 2). Our data also indicate that linezolid resistance rates in MRSA isolates were higher in isolates from ICUs compared to isolates from non-ICUs [0.56% (95% CI 0.36–0.87%) vs. 0.24% (95%CI 0.14–0.38%), *p* < 0.01], which was also confirmed by the multivariable analysis [adjusted OR: 2.86 (95% CI 1.58–5.19), *p* < 0.001] (Figure 2C, Table 3).

**TABLE 3:** Multivariable logistic regression analysis of factors associated with linezolid resistance in methicillin-resistant *S. aureus* blood isolates.

		*Multivariable analysis*		
		*OR*	*(95% CI)*	*p-value*
**Year of sampling (per 1 year increase)**				
	2014–2018	0.90	(0.70–1.16)	0.410
**Unit type**				
	Non-ICU	1	–	–
	ICU	2.86	(1.58–5.19)	<0.001
	Unknown	1.03	(0.27–3.97)	0.963
**European region**				
	Eastern	1	–	–
	Northern	0.97	(0.16–5.93)	0.977
	Western	0.93	(0.30–2.90)	0.903
	Southern	2.55	(0.94–6.89)	0.067
**Patient age**				
	<1 year	1	–	–
	1–19 years	0.27	(0.03–2.08)	0.208
	20–64 years	0.41	(0.09–1.90)	0.255
	≥65 years	0.34	(0.07–1.53)	0.159
	Unknown	0.56	(0.13–2.50)	0.451
**Patient gender**				
	Female	1	–	–
	Male	1.03	(0.54–1.99)	0.920
	Unknown	1.05	(0.49–2.22)	0.907

***OR:** Odds Ratio; **CI:** Confidence Interval; **ICU:** Intensive Care Unit.*

### Daptomycin Resistance in MRSA

**FIGURE 3:**
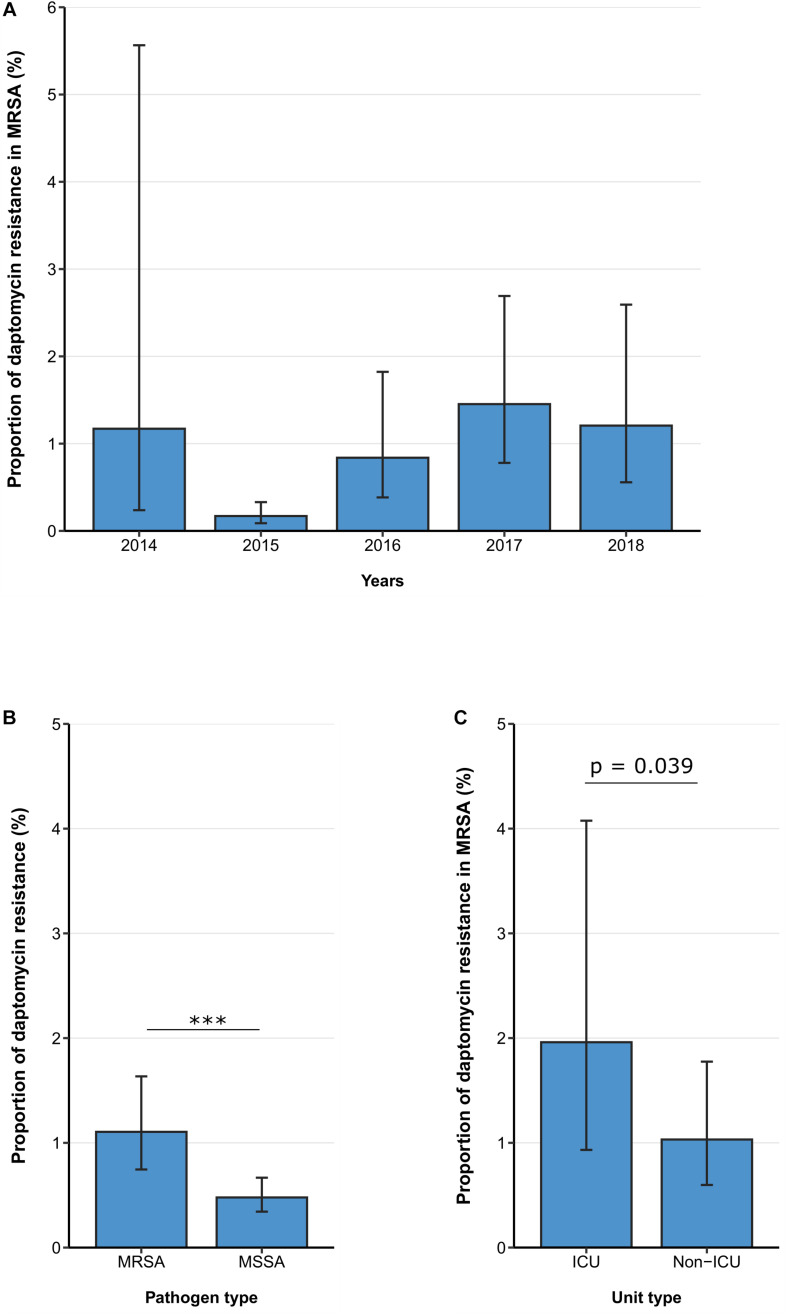
Daptomycin resistance in methicillin-resistant and -sensitive *S. aureus* isolates from patients with bloodstream infections. **(A)** Daptomycin resistance proportions in methicillin-resistant *S. aureus* (MRSA) between 2014 and 2018, **(B)** daptomycin resistance in MRSA and methicillin-sensitive *S. aureus* (MSSA), and **(C)** daptomycin resistance in MRSA stratified by unit type (ICU vs. non-ICU). Daptomycin resistance proportions are expressed as population-weighted mean proportions of resistant isolates among all tested isolates with corresponding 95% confidence intervals. **(B)** The difference in daptomycin resistance proportions between MRSA and MSSA isolates, and **(C)** between ICU vs. non-ICU MRSA isolates was analyzed using the Chi square test. *** *p* < 0.001.

Between 2014 and 2018, the daptomycin resistance proportion among MRSA isolates from patients with bloodstream infections was 1.11% (95% CI 0.75–1.63%) (Figure 3A) and no clear temporal trend was observed. [adjusted OR: 1.38 (95% CI 0.94–2.05%), *p* = 0.104]. Similar to the findings for linezolid, daptomycin resistance was higher among MRSA isolates than among methicillin-sensitive *S. aureus* isolates [1.11% (95% CI 0.75–1.63%) vs. 0.48% (95% CI 0.34–0.67%), *p* < 0.001] (Figure 3B), which was also confirmed by the multivariable regression analysis [adjusted OR: 2.25 (95% CI 1.45–3.49), *p* < 0.001]. There was a statistically significant higher daptomycin resistance proportion in MRSA isolates from ICUs compared to isolates from non-ICUs [1.96% (95% CI 0.93–4.08%) vs. 1.03% (95% CI 0.60–1.78%), *p* = 0.039] (Figure 3C). This finding was also observed after adjusting for other potential predictors in a multivariable regression analysis, that showed that there was a higher likelihood of daptomycin resistance in MRSA blood isolates from ICUs compared to non-ICU wards [adjusted OR: 2.65 (95% CI 1.13–6.26), *p* = 0.0245] (Table 4).

**TABLE 4:** Multivariable logistic regression analysis of factors associated with daptomycin resistance in methicillin-resistant *S. aureus* blood isolates.

		*Multivariable analysis*		
		*OR*	*(95% CI)*	*p-value*
**Year of sampling (per 1 year increase)**				
	2014–2018	1.38	(0.94–2.05)	0.104
**Unit type**				
	Non-ICU	1	–	–
	ICU	2.65	(1.13–6.26)	0.026
	Unknown	0.26	(0.08–0.84)	0.025
**European region**				
	Eastern	1	–	–
	Northern	3.96	(0.89–17.6)	0.072
	Western	2.93	(0.93–9.22)	0.067
	Southern	2.12	(0.88–5.13)	0.097
**Patient age**				
	<1 year	1	–	–
	1–19 years	2.60	(0.21–32.6)	0.458
	20–64 years	12.3	(1.26–120)	0.032
	≥65 years	7.81	(0.94–65.1)	0.058
	Unknown	14.1	(1.64–122)	0.016
**Patient gender**				
	Female	1	–	–
	Male	0.62	(0.24–1.65)	0.344
	Unknown	0.72	(0.31–1.70)	0.455

***OR:** Odds Ratio; **CI:** Confidence Interval; **ICU:** Intensive Care Unit.*

## Discussion

In this study, we examined linezolid and daptomycin resistance patterns among VREF and MRSA isolates isolated from patients with bloodstream infections in Europe. In sum, resistance proportions of the analyzed last line antibiotics in VREF and MRSA are relatively low in Europe. The population-weighted mean proportion of linezolid resistance among VREF isolates was 1.6% (95% CI 1.3–2.0%). While the in-country proportion varies across several local settings within Europe (Gawryszewska et al., 2016; Sassi et al., 2019; Xanthopoulou et al., 2020), this European mean proportion is comparably similar to what is seen in many within-country studies from other regions of the world such as South Korea, China, India, Iran, and United States where it remains less than 2% (Flamm et al., 2015; Houri et al., 2017; Yadav et al., 2017; Bi et al., 2018; Cho et al., 2018; Zhang et al., 2018; Huang et al., 2019).

Also, the European mean proportion of linezolid resistance among MRSA blood isolates included in this study was found to be very 1ow [0.29% (95% CI 0.21–0.40%)], attesting to the overall susceptibility of MRSA isolates to linezolid in Europe. This is also similar to local studies in Germany and Spain (Sierra et al., 2013; Yayan et al., 2015), Russia (Gostev et al., 2015), China (Huang et al., 2019), and Latin America (Vega and Dowzicky, 2017), but remarkably lower compared to results from Pakistan (48.1%) (Azhar et al., 2017) and India (2.8–7.0%) (Kaur and Chate, 2015; Kumar, 2016). In comparison to other multi-country surveillance studies, such as ZAAPS and SENTRY, linezolid activity against VREF and MRSA remains similarly very high (>98%) in Europe (Mendes et al., 2016; Deshpande et al., 2018).

Our results suggested no significant change in the linezolid resistance proportions among MRSA and VREF isolates over the study period in Europe despite the reported increasing trend of linezolid consumption between 2009 and 2018 in Europe (European Centre for Disease Prevention Control, 2019a; Kramer et al., 2019). We also found that VREF and MRSA isolates are more likely to be linezolid-resistant than the vancomycin-sensitive and methicillin-sensitive isolates, respectively. While previous studies have shown linezolid resistance can be seen in both VREF and vancomycin-sensitive isolates (Allen and Bierman, 2009), the increased likelihood among VREF and MRSA isolates suggests the higher chance of co-resistance to linezolid among VREF and MRSA isolates under selective pressure such as linezolid exposure (Cantón and Ruiz-Garbajosa, 2011). Whether this is driven mainly by *de novo* mutations and/or horizontal transfer of resistance genes is for future studies to clarify. However, this observation reinforces the need to preserve the effectiveness of vancomycin and penicillinase-resistant β-lactams, to reduce the use of linezolid and safeguard its efficacy as an important last-line antibiotic.

In line with the findings for linezolid resistance, the European population-weighted mean proportion of daptomycin resistance among MRSA isolates was similarly low [1.0% (95% CI 0.82–1.28%)]. The low proportion is comparable to the non-susceptibility proportion recorded in a multicenter ICU surveillance studies from Canada (Denisuik et al., 2018), China (Qin et al., 2016), and a global surveillance that reported daptomycin resistance in MRSA isolates of bone and joint infections (Jones et al., 2017). Similar to linezolid resistance, our results showed daptomycin resistance among MRSA isolates is substantially more likely compared to MSSA isolates. Our data showed MRSA blood isolates from the ICU are more likely to be linezolid and daptomycin resistant compared to isolates from non-ICU wards of the hospital. This might be due to the prominence of MRSA in the European ICUs (Vincent et al., 1995; Pujol et al., 1996; Boncagni et al., 2015; European Centre for Disease Prevention Control, 2018c) where linezolid and daptomycin are frequently prescribed antibiotic (Davis et al., 2005; Rodríguez et al., 2009; Hashemian et al., 2018; Kramer et al., 2019). These results can’t overstate the need for early initiation of effective antibiotics to treat MRSA infections especially among vulnerable patients that usually populate ICU wards, as a matter of patient safety.

Various mechanisms underlie the resistance of gram-positive pathogens to linezolid including mutation of 23S rRNA, ribosomal proteins (L3, L4) but very few studies have reported the transmissible *optrA*, *cfr*, and *poxtA* mediated linezolid resistance in Europe. The recent report of the highest prevalence (22.7%) of *optrA*, and *poxtA* genes ever in human linezolid-resistant enterococci isolates in Ireland (Egan et al., 2020), highlights its potential clinical and surveillance challenge in Europe (Feßler et al., 2013; Brenciani et al., 2015; Antonelli et al., 2016; de Dios Caballero et al., 2016; Gawryszewska et al., 2017; Lazaris et al., 2017; Bender et al., 2018a, b; Ruiz-Ripa et al., 2020, 2021).

Experience with the evolution of antibiotic resistance warns that the present effectiveness comes with a price of triggered treatment failure among future patients. With the treatment difficulties associated with VREF and MRSA, an increasing reliance on linezolid and daptomycin is expected since both have been shown to be equally efficacious in the treatment of VREF and MRSA (Crank et al., 2010; Gonzalez-Ruiz et al., 2011; Twilla et al., 2012; Heidary et al., 2018). Prevention of resistance to linezolid and daptomycin will require multi-pronged approaches including genomic surveillance, screening of high-risk patients where beneficial, combination antibiotic therapy, and enhanced infection prevention and control strategies (Gonzales et al., 2015; Yim et al., 2017). Therefore, the low proportion of resistance to linezolid and daptomycin among VREF and MRSA isolates in Europe should be seen as a window of opportunity to strengthen existing AMR prevention and control measures, and tackle the difficulties plaguing the research and development of new antibiotics (McKenna, 2020).

### Strengths and Limitations

With 7,000 and 35,000 VREF and MRSA isolates from patients with bloodstream infections, respectively, this study is the largest and most comprehensive analysis of last line antibiotic resistance profiles among these difficult to treat multidrug resistant pathogens in the EU/EEA. The analyzed dataset shows a high level of representativeness for the EU/EEA region, since the isolates were derived from routine clinical microbiological sampling and antimicrobial susceptibility testing and were transmitted to EARS-Net from 29 EU/EEA countries and the United Kingdom. Notably, population, hospital, and isolate sample representativeness was assessed as “high” in 25 countries (European Centre for Disease Prevention Control, 2019b). In addition, the validity of these AMR data is regularly assessed in external quality assessments of participating laboratories (European Centre for Disease Prevention Control, 2018b) ensuring high data quality.

A potential limitation is the variation in population coverage among reporting countries, although half of the participating countries reported a national coverage greater than 80% (European Centre for Disease Prevention Control, 2019b). To minimize possible bias from differences in population size and isolate numbers from various countries, EU/EEA-wide resistance proportions and multivariable regression analyses were population-weighted based on the population sizes of the individual regions. Moreover, differences in sampling routines, admission characteristics (e.g., stay duration, bed space density) and healthcare resources across EU/EEA countries can result in biased estimates of resistance proportions. However, to reduce the extent of this potential bias, in EARS-Net only isolates from bloodstream infections are collected, since microbiological sampling routines for these infections are generally similar across countries of the EU/EEA, even though some variations (such as frequency of sampling) between hospitals and countries cannot fully excluded.

Also, despite the off-label use of linezolid as a therapy option in some settings, it is questionable whether blood isolates are the most appropriate to determine linezolid resistance among VREF isolates from patient with nosocomial bloodstream infections because of its non-bactericidal effects. Therefore, our results should be interpreted with these limitations in mind.

## Conclusion

Proportions of linezolid and daptomycin resistance among VREF and MRSA blood isolates remain low in the EU/EEA throughout the study period. However, VREF and MRSA were consistently more resistant to linezolid and daptomycin compared to their sensitive isolates. MRSA blood isolates from the ICU were more likely to be linezolid and daptomycin resistant compared to isolates from non-ICU wards of the hospital. In addition to existing antibiotics stewardship programs, it is necessary to strengthen surveillance in Europe, especially the genomic characterization of the resistance genes that compromise the efficacy of these last line antibiotics in VREF and MRSA.

## Data Availability Statement

The data analyzed in this study is subject to the following licenses/restrictions: The datasets will be made available with the express permission of the European Centre for Disease Control (ECDC) Stockholm Sweden. The ECDC on behalf of the EU/EEA countries have the exclusive control over the sharing of the data. Requests to access these datasets should be directed to DATA ACCESS REQUEST European Centre for Disease Prevention and Control (ECDC) Gustav III:s boulevard, 40 16973 Solna Sweden. Further inquiries should be directed to the corresponding author.

## Author Contributions

OA, RM, and TE: conceptualization. RM and OA: original draft. NW, RM, and OA: statistical analysis. RM, OA, GW, and TE: critical review, revision, and Editing. RM, OA, NW, GW, and TE: final approval. All authors contributed to the article and approved the submitted version.

## Conflict of Interest

The authors declare that the research was conducted in the absence of any commercial or financial relationships that could be construed as a potential conflict of interest.

## Abbreviations

BSI: bloodstream infections
CDC: Center for Disease Control and Prevention
ECDC: European Centre for Disease Prevention and Control
HAIs: hospital-acquired infections
E: *Enterococcus faecium*
VREF: vancomycin-resistant *Enterococcus faecium*
MRSA: Methicillin-resistant Staphylococcus aureus
LRVREF: Linezolid-Vancomycin resistant *Enterococcus faecium*
ICU: intensive care unit
NHSN: National Healthcare Safety Network
WHO: World Health Organization
95% CI: 95% confidence interval
ZAAPS: Zyvox^§^ Annual Appraisal of Potency and Spectrum programme
SENTRY: SENTRY Antimicrobial Surveillance Program
PCR: Polymerase Chain Reaction.
