# Supplementary material for: Low Proportion of Linezolid and Daptomycin Resistance Among Bloodborne Vancomycin-Resistant Enterococcus faecium and Methicillin-Resistant Staphylococcus aureus Infections in Europe
Source: Front Microbiol. 2021 May 31;12:664199. doi: 10.3389/fmicb.2021.664199 (PMC8203336; doi:10.3389/fmicb.2021.664199)
Supplement: Supplementary file 1 [file Table_1.docx]

**Supplementary Table 1.** Multivariable logistic regression analysis of factors associated with linezolid resistance in *E. faecium* blood isolates

|  |  | ***Multivariable analysis*** | | |
| --- | --- | --- | --- | --- |
|  |  | ***OR*** | ***(95% CI)*** | ***p-value*** |
| ***Year of sampling (per 1year increase)*** | |  |  |  |
|  | 2014-2018 | 0.92 | (0.84-1.01) | 0.077 |
| ***Pathogen type*** | |  |  |  |
|  | VSEF | 1 | - | - |
|  | VREF | 1.99 | (1.56-2.54) | <0.001 |
| ***Unit type*** | | | | |
|  | Non-ICU | 1 | - | - |
|  | ICU | 1.12 | (0.86-1.46) | 0.393 |
|  | Unknown | 1.68 | (1.08-2.61) | 0.020 |
| ***European region*** | |  |  |  |
|  | Eastern | 1 | - | - |
|  | Northern | 0.75 | (0.43-1.29) | 0.292 |
|  | Western | 1.07 | (0.70-1.65) | 0.755 |
|  | Southern | 2.33 | (1.50-3.63) | <0.001 |
| ***Patient age*** | |  |  |  |
|  | <1 year | 1 | - | - |
|  | 1-19 years | 1.44 | (0.39-5.29) | 0.587 |
|  | 20-64 years | 1.04 | (0.33-3.31) | 0.945 |
|  | ≥65 years | 0.68 | (0.21-2.14) | 0.503 |
|  | Unknown | 0.88 | (0.22-3.54) | 0.858 |
| ***Patient gender*** | |  |  |  |
|  | Female | 1 | - | - |
|  | Male | 1.05 | (0.85-1.31) | 0.650 |
|  | Unknown | 1.06 | (0.69-1.64) | 0.781 |

**OR**: Odds Ratio; **CI**: Confidence Interval; **ICU**: Intensive Care Unit
