# Supplementary material for: Low Proportion of Linezolid and Daptomycin Resistance Among Bloodborne Vancomycin-Resistant Enterococcus faecium and Methicillin-Resistant Staphylococcus aureus Infections in Europe
Source: Front Microbiol. 2021 May 31;12:664199. doi: 10.3389/fmicb.2021.664199 (PMC8203336; doi:10.3389/fmicb.2021.664199)
Supplement: Supplementary file 2 [file Table_2.docx]

**Supplementary Table 2.** Multivariable logistic regression analysis of factors associated with linezolid resistance in *S. aureus* blood isolates

|  |  | ***Multivariable analysis*** | | |
| --- | --- | --- | --- | --- |
|  |  | ***OR*** | ***(95% CI)*** | ***p-value*** |
| ***Year of sampling (per 1year increase)*** | |  |  |  |
|  | 2014-2018 | 1.05 | (0.90-1.23) | 0.556 |
| ***Pathogen type*** | |  |  |  |
|  | MSSA | 1 | - | - |
|  | MRSA | 2.74 | (1.98-3.78) | <0.001 |
| ***Unit type*** | | | | |
|  | Non-ICU | 1 | - | - |
|  | ICU | 1.99 | (1.36-2.90) | <0.001 |
|  | Unknown | 0.99 | (0.56-1.73) | 0.959 |
| ***European region*** | |  |  |  |
|  | Eastern | 1 | - | - |
|  | Northern | 1.42 | (0.59-3.46) | 0.436 |
|  | Western | 1.11 | (0.54-2.26) | 0.774 |
|  | Southern | 3.23 | (1.52-6.83) | 0.002 |
| ***Patient age*** | |  |  |  |
|  | <1 year | 1 | - | - |
|  | 1-19 years | 0.67 | (0.20-2.19) | 0.503 |
|  | 20-64 years | 0.60 | (0.25-1.41) | 0.240 |
|  | ≥65 years | 0.78 | (0.35-1.78) | 0.559 |
|  | Unknown | 0.99 | (0.33-3.01) | 0.988 |
| ***Patient gender*** | |  |  |  |
|  | Female | 1 | - | - |
|  | Male | 0.85 | (0.60-1.21) | 0.361 |
|  | Unknown | 1.26 | (0.70-2.27) | 0.433 |

**OR**: Odds Ratio; **CI**: Confidence Interval; **ICU**: Intensive Care Unit
