# Supplementary material for: Low Proportion of Linezolid and Daptomycin Resistance Among Bloodborne Vancomycin-Resistant Enterococcus faecium and Methicillin-Resistant Staphylococcus aureus Infections in Europe
Source: Front Microbiol. 2021 May 31;12:664199. doi: 10.3389/fmicb.2021.664199 (PMC8203336; doi:10.3389/fmicb.2021.664199)
Supplement: Supplementary file 3 [file Table_3.docx]

**Supplementary Table 3.** Multivariable logistic regression analysis of factors associated with daptomycin resistance in *S. aureus* blood isolates

|  |  | ***Multivariable analysis*** | | |
| --- | --- | --- | --- | --- |
|  |  | ***OR*** | ***(95% CI)*** | ***p-value*** |
| ***Year of sampling (per 1year increase)*** | |  |  |  |
|  | 2014-2018 | 0.99 | (0.76-1.30) | 0.963 |
| ***Pathogen type*** | |  |  |  |
|  | MSSA | 1 | - | - |
|  | MRSA | 2.25 | (1.45-3.49) | <0.001 |
| ***Unit type*** | | | | |
|  | Non-ICU | 1 | - | - |
|  | ICU | 2.17 | (0.97-4.86) | 0.606 |
|  | Unknown | 1.57 | (0.68-3.63) | 0.289 |
| ***European region*** | |  |  |  |
|  | Eastern | 1 | - | - |
|  | Northern | 1.45 | (0.64-3.28) | 0.375 |
|  | Western | 1.37 | (0.75-2.50) | 0.313 |
|  | Southern | 1.35 | (0.84-2.17) | 0.221 |
| ***Patient age*** | |  |  |  |
|  | <1 year | 1 | - | - |
|  | 1-19 years | 8.39 | (1.86-37.7) | 0.006 |
|  | 20-64 years | 6.83 | (1.83-25.6) | 0.004 |
|  | ≥65 years | 5.25 | (1.52-18.1) | 0.009 |
|  | Unknown | 5.33 | (1.41-20.1) | 0.014 |
| ***Patient gender*** | |  |  |  |
|  | Female | 1 | - | - |
|  | Male | 0.62 | (0.38-1.0) | 0.048 |
|  | Unknown | 1.07 | (0.56-2.05) | 0.845 |

**OR**: Odds Ratio; **CI**: Confidence Interval; **ICU**: Intensive Care Unit
